# Evaluation of the predictive value of tonsil examination by bacteriological culture for detecting positive lung colonization status of nursery pigs exposed to *Actinobacillus pleuropneumoniae* by experimental aerosol infection

**DOI:** 10.1186/s12917-018-1542-9

**Published:** 2018-06-28

**Authors:** Doris Hoeltig, Florian Nietfeld, Katrin Strutzberg-Minder, Judith Rohde

**Affiliations:** 1 Clinic for Swine and Small Ruminants, Forensic Medicine and Ambulatory Service, University of Veterinary Medicine, Foundation, Bischofsholer Damm 15, D-30173 Hannover, Germany; 2 Institute for Microbiology, University of Veterinary Medicine, Foundation, Bischofsholer Damm 15, D-30173 Hannover, Germany; 3 Clinic for Swine, Department of Veterinary Medicine, Justus-Liebig-University Giessen, Frankfurter Str. 112, D- 35392 Giessen, Germany; 4 Innovative Veterinary Diagnostics (IVD-GmbH), Albert-Einstein-Str. 5, 30926 Seelze, Germany

**Keywords:** Detection, Sensitivity, Specificity, *A. pleuropneumoniae*, Culture method

## Abstract

**Background:**

*Actinobacillus (A.) pleuropneumoniae* is the causative agent of porcine pleuropneumonia. For control of the disease the detection of sub-clinically infected pigs is of major importance to avoid transmitting of subclinical infections. One method recommended is the testing of tonsillar samples for the presence of *A. pleuropneumoniae*. This is routinely done by PCR techniques. However, based upon PCR susceptibility testing and monitoring of resistance development is impossible. Therefore, in this study the informative values of bacteriological culture of tonsilar samples for the colonisation status of pigs were tested. In total, 163 German Landrace nursery pigs were experimentally exposed to *A. pleuropneumoniae* serotype 7 by aerosol and the rate of isolation from lung tissue and tonsils and the corresponding degree of lung lesions were investigated.

**Results:**

Overall a significant correlation (*p* < 0.001) between degree of clinical disease, degree of lung alterations and degree of *A. pleuropneumoniae* isolation from tonsillar and lung tissue after exposure was detected. Of these animals tested, 74.8% were tested positive in tonsillar and lung samples, 7.4% remained completely negative and in 4.3% the tonsils were tested positive despite negative isolation results from lung tissue. In 13.5% of the pigs *A. pleuropneumoniae* could be isolated in lung tissue but not in tonsillar samples. In 36.4% of these animals a heavy colonization of the lungs and in 40.9% moderate to severe lung alterations were proven. Hence, the diagnostic sensitivity for the detection of a positive colonization status of the pigs by bacterial culture examination of tonsillar samples was 84.7%, the diagnostic specificity was 66.7% and the predictive values were 94.6% (positive) and 35.3% (negative). The overall sensitivity for *A. pleuropneumoniae* exposure was 78.2% (tonsils) and 88.0% (lung tissue).

**Conclusions:**

In conclusion, tonsil examination alone for the detection of a positive colonization status of pigs performed might lead to false negative results as lungs might be heavily colonized despite negative tonsillar isolation results. Therefore culture of tonsillar samples should not be the sole test for the confirmation of a pigs’ status but used in combination with methods also evaluating the colonization status of the lower respiratory tract.

## Background

*Actinobacillus* (*A.*) *pleuropneumoniae* is the causative agent of porcine pleuropneumia, a respiratory disease of pigs causing severe economic losses in pork production worldwide. The infection of pigs can lead to subclinical infection without any clinical signs of disease, peracute death, acute respiratory disease which is often accompanied by high mortality and chronic disease leading to recurrent coughing and wasting of the animals [1]. In cases of subclinical infection, often only the oropharyngeal cavity of the pigs is colonized [2]. Apart from tonsils, *A. pleuropneumoniae* is also able to survive in lungs and the affected pigs may appear clinically healthy. Additionally, even antibiotic treatment is not able to clear *A. pleuropneumoniae* completely from these localizations [3]. These subclinically infected pigs are the major sources for spreading of the disease, either by transmission of the pathogen from the sows to their offspring or by pig-to-pig contact during trading of living animals [4]. Studies show that more than 30% of piglets from infected sows are positive for *A. pleuropneumoniae* at the time of weaning, increasing to 50% or more at the age of 10 weeks [5, 6].

Several methods are described for the detection of these carrier pigs. For herd screening different serological tests are used routinely. However, serology has some disadvantages as seroconversion generally occurs 10 to 14 days after infection [1]. Additionally, *A. pleuropneumoniae* can colonize the upper respiratory tract without inducing a seroconversion [7, 8]. Moreover, it is noteworthy that the presence of specific antibodies is not able to totally clear *A. pleuropneumoniae* totally from the upper respiratory tract of the colonized pigs [1]. In cases with inconclusive serological results or cases where *A. pleuropneumoniae* is expected to be present in very low numbers attempts for the direct detection of the pathogen are being made [6]. For identifying sub-clinically infected herds the direct detection of *A. pleuropneumoniae* from tonsils is recommended. This direct bacterial detection can be based on bacteriological culture [8, 9] or molecular detection of the pathogen by several PCR techniques [10, 11]. PCR techniques might seem to be more appropriate as they can also detect low numbers of pathogen DNA from viable and non-viable pathogens, whereas the isolation by bacteriological culture depends on the viability of the bacteria [10]. Another advantage is that PCR techniques do not bear the risk of overgrowth of the specific pathogen by the abundant microflora as the upper respiratory tract is heavily colonized by different bacterial species [12]. However, a major disadvantage of PCR detection, especially in the area of antimicrobial stewardship is that susceptibility testing is only possible from cultured bacteria so far. As an alternative for classical bacterial isolation, the immunomagnetic isolation of *A. pleuropneumoniae* [13, 14] is still very expensive and time-consuming. Thus the classical isolation of *A. pleuropneumoniae* by bacteriological culture from tonsillar samples retains importance.

As the transfer of the pathogen can either be from the sow to the offspring or by horizontal pig-to-pig contact [4] in this study tonsillar samples from experimentally infected nursery pigs were examined as nursery pigs are an age group often regrouped with a high risk of pathogen transfer. The tonsillar samples were investigated regarding applicability and the informative value of bacterial culture evaluating the hypothesis that the examination of tonsillar samples is an adequate tool to securely determine the colonization status of pigs in case of unclear serological results or recently suspected exposure.

## Methods

For this investigation tonsils and samples from lung tissue were taken from 163 pigs experimentally infected with *A. pleuropneumoniae* serotype 7. All pigs belonged to a commercial German Landrace breeding line, vaccinated against *Mycoplasma hyopneumoniae* and PCV-2 and were delivered from the same piglet producing farm considered negative for *A. pleuropneumoniae*. Regarding sex 52 pigs were female and 111 pigs were male-castrated and all pigs were tail docked and considered clinically healthy on the day of delivery. The pigs were kept under standardized level 2 conditions in accordance with the Guidelines for Protection of Vertebrate Animals used for experimental and other Scientific Purposes, European Treaty Series, nos. 123 /170 (http://conventions.coe.int/treaty/EN/treaties/html/123.htm; http://conventions.coe.int/treaty/EN/treaties/html/170.htm). Study design and housing of the animals were approved by a local, independent committee on ethics (Commission for ethical estimation of animal research studies of the Lower Saxonian State Office for Consumer Protection and Food Safety; approval number: 33.12–42,502–04-15/1962). The pigs were kept on concrete floor with 8m^2^ per 8 to 10 pigs. An area of 2.8m^2^ of each stable was covered with a rubber mat to provide a more comfortable bedding area. This area was additionally heated by two infrared lamps. Allocation to the housing groups was performed by simple randomization [15]. Ambient temperature was 27.5 °C ± 1.9 °C (MEAN ± SD) and humidity was 33.2% ± 12.5% (MEAN ± SD). The pigs were fed a commercial standardized diet and each housing group was provided 2.5 kg of hay flakes (®AGROBS Pre Alpin Wiesenflakes, Co. AGROBS, Degerndorf, Germany) per day as material for rooting and manipulation. Potable water was constantly available for free choice and of drinking water quality. The pigs arrived three weeks prior to infection for an adequate adaptation to diet, new environment and clinical examination procedures. The pigs were seven weeks of age at the time of exposure (SD: ±1.5 days). They had an average body weight of 12.0 kg (± 1.9 kg).

### Experimental exposure

Prior to experimental infection all pigs underwent a general clinical examination as well as a radiographic (80 kV no-scatter grid and automatic exposure; Precimat, Co. Picker International, Munich, Germany) and sonographic (8 MHz linear scanner, LOGIQ™ Book XP, Co. GE Medical Systems, Chalfont St. Giles, Great Britain) examination of the lungs. Radiography was performed in two views (latero-lateral and dorso-ventral). The film-focus distance for the x-ray examination was 1.5 m. Images were recorded with 240 × 300 mm image plate cassettes (Co. EURAS MedTech, PROVOTEC, X-RAY, Espelkamp, Hannover, Germany) and readout linearly with a digital non-contact image storage system (iCR 3600®, Co. EURAS MedTech, PROVOTEC, X-RAY, Espelkamp, Hannover, Germany). Bronchoalveolar lavage fluids (100 ml 0.9% NaCl-solution ad us. vet., Co. WDT, Garbsen, Germany) as well as serum samples were taken. The bronchoalveolar lavage fluid was examined by bacteriological culture and PCR [16] for the absence of *A. pleuropneumoniae*. Bronchoalveolar Lavage, radiographic and sonographic examination were performed under general anesthesia with 20 mg/kg Ketamine i.m. (Ketamin 100 mg/ml®, Co. CP-Pharma, Burgdorf, Germany) and 2 mg/kg Azaperon i.m. (Stresnil®, Co. Janssen-Cilag GmbH, Baar, Switzerland). Neuroleptanalgesia based upon ketamine and azaperon was chosen as this is the only official licensed anesthesia protocol for pigs in Germany. Serum samples were analyzed by ApxIV-ELISA (IDEXX APP-ApxIV Ab Test®, Co. IDEXX Laboratories, Maine, USA). All pigs were confirmed as clinically healthy and negative for *A. pleuropneumoniae* by direct and indirect screening procedures. This study was conducted as an uncontrolled exposure study; the experimental unit was the individual animal. The pigs were infected in groups of five or six pigs; each housing group was divided into two exposure groups. The assignment to the different infection groups was based upon simple randomization. In total the infection was independently repeated for 28 groups of pigs. The experimental procedure was identical for each exposure group. The experimental infection was conducted as an aerosol exposure with a total exposure time of 30 min [17]. Approximately 1 × 10^5^ bacteria of *A. pleuropneumoniae* AP76 serotype 7 were nebulized resulting in an aerosol concentration of 1 × 10^2^ colony forming units (cfu) per liter aerosol. After the aerosol exposure the pigs were clinically monitored every second hour for the first 48 h after exposure, and thereafter twice a day. Rescue criteria were defined to minimize the suffering of the infected pigs. These rescue criteria were a breathing frequency > 70/min and cyanosis and open-mouth breathing, apathy with no reactions to stimulation and standing with head down without lying down and vomiting as well as foam around nostrils or mouth, a rectal temperature of > 42.0°Cor < 37.5 °C, a body temperature > 40.3 °C in combination with a breathing frequency > 46/min and dyspnea as well as decreased feed intake for more than two consecutive days, a body temperature > 40.3 °C and decreased general condition in combination with a breathing frequency > 35/min for more than two consecutive days from day three after exposure; any non-predictable event, reaction to treatment or disease leading to a moderate degree of general condition reduction or pain for more than 48 h; any non-predictable event, reaction to treatment or disease leading to high degree of general condition reduction or pain.

### Necropsy and bacteriological examination

Seven days after infection or earlier in case of withdrawal due to rescue criteria the pigs were euthanized by intravenous injection of 80 mg/kg pentobarbital (Euthadorm®, Co. CP Pharma GmbH, Burgdorf, Germany). A necropsy was performed and the degree of developed lung lesions was assessed using a lung lesion score [18]. Each lung lobe could reach a maximum score of 5 resulting in an overall maximum score of 35. Additionally, samples for the isolation of *A. pleuropneumoniae* were taken from eight different localizations. In total, seven lung tissue samples (approximately 1 cm^2^) of defined positions located in the outer third of each of the seven lung lobes as well as the palatine tonsils were collected.

Samples were plated on *A. pleuropneumoniae*-selective blood agar [19] using the quadrant streaking method. Abundance of growth was assessed semi-quantitatively. Bacterial isolates were identified as *A. pleuropneumoniae* by PCR amplification of the apxIV gene [16].

The level of isolation from the lungs was translated into a isolation score [20]. The amount of growth for each localization (0 = no growth; 1 = sparse growth; 2 = moderate growth; 3 = heavy growth) was added and divided by the total number of lung tissue samples, results indicating the level of the isolation from lung tissue (0–1: low-grade; > 1–2: moderate, > 2–3: high-grade).

### Statistical methods

The collected data were transferred to a database based upon Excel ® (Co. Microsoft Cooperation, Dublin, Irland). Verification was assured by double data entry. Statistical analyses were carried out using Excel® and IBM SPSS Statistics® (Co. IBM Deutschland GmbH, Ehningen, Germany). For all continuous variables, sample size, mean (m), standard deviation (SD), median, quartiles, minimum and maximum were calculated. Categorical variables were displayed as absolute and relative frequencies. The applied level of significance was 5% (*p* < 0.05). For correlation analysis Spearman Rank Correlation was calculated. Primary criterion for the evaluation was the bacteriological re-isolation score of the whole lung. Secondary criteria for the analysis were the re-isolation score from different lung lobes, the lung lesion score and the severity of clinical symptoms.

## Results

After infection 39 of the 163 pigs developed no or only slight signs of acute porcine pleuropneumonia, 89 pigs showed moderate symptoms of disease and 35 pigs had to be euthanized prior to day 7 after exposure due to the severity of clinical signs and the defined rescue criteria. No overgrowth by accompanying microflora appeared and all bacteriological cultures could be evaluated.

The lung lesion score (LLS) was 0 for 14 animals and 35 for 12 animals. Regarding the isolation of *A. pleuropneumoniae* it could be isolated from the tonsils of 129 pigs (ts+) and from the lungs, it was isolated of 144 pigs (lg+). In 7 cases of isolation from the tonsils the lungs of the pigs remained negative (ts+/lg-). In case of isolation from the lungs was isolated to the highest degree from the caudal lobes (Table 1). Isolation was significantly higher from these lung lobes (*p* < 0.0001) compared to all other lung lobes. There was also a difference (*p* = 0.004) between the right cranial lung lobe and the right middle lobe of the lung with significantly (p = 0.004) higher level of isolation from the middle lobe compared to the cranial lobe of the right lung. In 12 pigs, *A. pleuropneumoniae* could not be isolated neither from the lungs nor from the tonsils (ts−/lg-). 19 pigs (ts+/lg-;*n* = 7 and ts−/lg-;*n* = 12) were euthanized at the end of the trial, none of these pigs had to be euthanized due to rescue criteria .

**Table 1:** Distribution of *A. pleuropneumoniae* colonization status by bacteriological culture and corresponding clinical, pathomorphological and bacteriological examination results

	Detected *Actinobacillus pleuropneumoniae* status by bacteriological culture (*n* = 163)			
	Tonsils: negative Lungs: negative	Tonsils: negative Lungs: positive	Tonsils: positive Lungs: negative	Tonsils: positive Lungs: positive
Total number of animals	12	22	7	122
Degree of clinical disease^a^ *(number of animals)*				
- No disease	8	0	4	4
- Mild disease	4	3	3	13
- Moderate disease	0	13	0	76
- Severe Disease	0	6	0	29
Euthanized due to Rescue criteria *(number of animals)*	0	6	0	29
Lung lesions score^a^ *(LLS; number of animals)*				
- No lesions	5	0	4	4
- LLS: 0.1–12.5	6	13	3	49
- LLS: 12.5–25	1	7	0	41
- LLS: > 25	0	2	0	28
Degree of isolation from lung tissue^a^ *(number of animals)*				
- No isolation	12	0	7	0
- Low-grade	0	7	0	16
- moderate	0	7	0	51
- high-grade	0	8	0	55
Positive test results for different lung lobes (%)				
- Cranial lobes	0	76,2	0	93,0
- Middle lobes	0	76,2	0	88,5
- Caudal lobes	0	83,3	0	93,2
- Lobus accessorius	0	85,7	0	91,8

^a^significant correlation between lung lesion score, overall isolation from lungs and overall isolation from tonsils

Only 84.7% of the 144 pigs carrying *A. pleuropneumoniae* in the lungs were detected by bacteriological examination of the tonsils leaving 22 pigs positive for *A. pleuropneumoniae* (ts−/lg+) undetected. From the 15.3% of the pigs where *A. pleuropneumoniae* was not detected by tonsil examination one pig was tested positive in one lung lobe, two pigs in two lung lobes and one pig in three lung lobes. Three pigs had positive isolation results in the samples from five lung lobes, five pigs in samples from six lung lobes and in 10 pigs all lung lobes were tested positive for A*. pleuropneumoniae* by bacteriological examination. Of these ts−/lg + pigs 31.8% had an isolation score ≤ 1, 27.3% had a score between > 1 and ≤ 2 and 40.9% a score > 2 and ≤ 3. Six animals where *A. pleuropneumoniae* was detected in the lungs but not on the tonsils were euthanized because of the severity of symptoms prior to day 7 post infection and 16 animals were euthanized at the end of the trial .

Comparing the lung lesion score and the isolation results, *A. pleuropneumoniae* could be isolated from the lungs of all pigs showing LLS of 35 (*n* = 12), but only from the tonsils of 10 of these pigs. Within the group of pigs showing a LLS of 0 (*n* = 14), *A. pleuropneumoniae* could be isolated from the tonsils of 8 pigs but only from the lungs of 3 pigs.

A statistically significant correlation between lung lesion score, isolation from the lungs and isolation from the tonsils was detected (*p* < 0.001). The sensitivity for the detection of a colonization status of the pig by bacteriological examination of the tonsils was 84.7%, the specificity was 63.2%, the positive predictive value was 94.6%, and the negative predictive value was 35.3% (Table 2). The rate of detection of the previous *A. pleuropneumoniae* exposure of the animals was 79.1% for the tonsil examination and 88.3% for the examination of the lung tissue.

**Table 2:** 2 × 2 contingency table for statistical calculation of SEN, SP, PPV and NPV for the detection of a positive *A. pleuropneumoniae* lung colonization status by bacteriological culture examination of tonsilar samples (*n* = 163)

	Samples positive by lung tissue examination	Samples negative by lung tissue examination
Samples positive by tonsil examination	122 (true positive)	7 (false positive)
Samples negative by tonsil examination	22 (false negative)	12 (true negative)
Sensitivity (SEN)	true positive / (true positive + false negative) =	84.72%
Specificity (SP)	true negative / (true negative + false positive) =	63.16%
Positive predictive value (PPV)	true positive /(true positive + false positive)=	94.57%
Negative predictive value (NPV)	True negative / (true negative + false negative) =	35.29%

## Discussion

As the exposed pigs showed the typical spectrum of clinical signs of porcine pleuropneumonia including death, as well as subclinical infection without any clinical signs of disease, the exposure protocol can be considered successful. As the evaluation of all cultures from tonsillar samples was possible and no sample had to be excluded due to overgrowth by accompanying microflora this study shows that in contrast to the results of other studies [11, 13] the bacteriological culture using selective media [18] can be utilized for the detection of *A. pleuropneumoniae* in tonsillar samples. Nevertheless, resulting isolates should be confirmed by PCR [15] to avoid an incorrect classification of bacteria that are highly related to *A. pleuropneumoniae* [12].

Our isolation results confirm the findings of previous studies [2] that after an *A. pleuropneumoniae* infection the pathogen may still be present on the tonsils despite being cleared from the lung tissue by the host defense mechanisms. However, we also showed that the opposite may be possible, *A. pleuropneumoniae* may colonize the lungs without a detectable colonization of the tonsillar crypts. That *A. pleuropneumoniae* was not detected in some tonsillar samples may be due to the amount of living bacteria within the samples being too low to gain a positive result by bacteriological culture, and perhaps may have been detected using PCR protocols [10]. However, PCR results would also not have given insight to the viability of the *A. pleuropneumoniae* isolates as it also detects non-viable pathogen DNA and an antibiotic sensitivity testing would not be possible. As these ts-lg + results not only appeared in pigs evaluated at the end of the trial but also in animals that had been euthanized due to the severity of symptoms we would expect to be able to re-isolate from tonsils. That this was not possible from some pigs suggests they present a risk of transferring the bacteria to other pigs. These pigs would have been detected only if the tonsillar examination recommended for the detection of sub-clinically infected pigs [6, 8, 9, 13] had been combined with diagnostic techniques also examining the status of the lower respiratory tract.

Under field condition, especially in cases of the examination of trading pigs alive, a sampling of seven lung tissue samples, one from each lung lobe, in most cases is not practicable. A method developed for the examination of the bacterial colonization of the lungs from living animals is the bronchoalveolar lavage [21]. However it should be taken into account that information on detection rates of *A. pleuropneumoniae* from bronchoalveolar lavage (BAL) in comparison to lung tissue is sparse. The level of isolation of *A. pleuropneumoniae* was highest for the diaphragmatic lobes and these lobes are the main localizations reached by BAL without visual control [21, 22]. Therefore, it is reasonable to assume that colonization of the lung would have been detected by culturing BAL.

The observed correlation between isolation from the tonsils, isolation from the lung tissue and degree of lung lesions might lead to the assumption that the examination of the tonsils would be representative for the status of the whole respiratory tract regarding *A. pleuropneumoniae* colonization. Nevertheless, the negative predictive value was quite low. However, as our results are based upon an experimental exposure where all pigs presumably had contact with *A. pleuropneumoniae,* the prevalence of *A. pleuropneumoniae* colonization in this population might be higher than in a field population of swine. If so, with a lower prevalence of *A. pleuropneumoniae* infected animals in a field population, the negative predictive value of the culture of tonsillar samples would increase while the positive predictive value would decrease [23–25]. Another fact that might change the results under field conditions is the involved serotype of *A. pleuropneumoniae*. In this study only one strain of *A. pleuropneumoniae* serotype 7 was tested. Different factors are involved in the adhesion of *A. pleuropneumoniae* to the porcine respiratory tract including fimbrial structures, lipopolysaccharides, outer membrane proteins as well as genes involved in biofilm formation. Appearance and molecular structure of these virulence factors can be different between different serotypes and strains of the same serotypes of *A. pleuropneumoniae*. Additionally, their involvement in the adhesion process has mainly been shown for the lower respiratory tract and information on virulence factors responsible for the colonization of the upper respiratory tract is scarce [26]. Nevertheless such differences in virulence factors of the involved serotypes may change the test results under field conditions as often several strains of *A. pleuropneumoniae* are present in the same herd [27] and may also colonize the respiratory tract of the same pig.

## Conclusions

In conclusion, bacteriological culture of tonsillar samples can be a useful tool for the detection of *A. pleuropneumoniae* if information on viability and antibiotic sensitivity of the isolates is needed. However, especially in herds with high traffic of animals or when trading of animals with a SPF status, culture of tonsils should not be performed as the only method for the detection of sub-clinically infected pigs. In cases of unclear serological results or if the possible date of infection is considered less than 10 days, the culturing of the tonsils should not be performed solely for the status evaluation of the pigs due to the low estimated negative predictive value of the tonsillar examination and the overall low sensitivity of the tonsillar examination regarding previous *A. pleuropneumoniae* contact.

## Abbreviations

A. pleuropneumoniae: Actinobacillus pleuropneumoniae
BAL: Bronchoalveolar lavage
Co: Company
ELISA: Enzyme-Linked Immunosorbent Assay
i.m.: intramuscular
kg: kilogram
kV: kilo voltage
lg: lung
LLS: Lung Lesion Score
mg: milligram
min: minutes
ml: milliliter
NPV: Negative predictive value
p.inf.: post infectionem
PCR: Polymerase chain reaction
PPV: Positive predictive value
SEN: Sensitivity
SPF: Specific pathogen free
ts: Tonsillar
